# Controlled Exit from the G2/M Checkpoint in RPE-1 Cells Using RO3306: Enrichment of Phase-Specific Cell Populations for In-Depth Analyses of Mitotic Events

**DOI:** 10.3390/ijms26104951

**Published:** 2025-05-21

**Authors:** Teresa Anglada, Núria Pulido-Artola, Marina Rodriguez-Muñoz, Anna Genesca

**Affiliations:** Department of Cell Biology, Physiology, and Immunology, Universitat Autònoma de Barcelona, 08193 Barcelona, Spain; nuria.pulido@uab.cat (N.P.-A.); marina.rodriguez.munoz@uab.cat (M.R.-M.)

**Keywords:** cell division, mitosis, RO3306, synchronization, cell cycle

## Abstract

Studying the cell cycle is essential for understanding the molecular mechanisms that regulate cell division, growth, and differentiation in living organisms. However, mitosis constitutes only a brief phase of the overall cell cycle, making its analysis challenging in asynchronous cell populations due to its transient and dynamic nature. Cell synchronization methods help to enrich populations at specific cell cycle stages, including mitosis, typically by using chemical inhibitors to arrest cells at defined checkpoints. However, many existing protocols rely on combinations of inhibitors that interfere with normal mitotic progression, disrupting dynamics and causing side effects such as chromosome non-disjunction or lagging chromosomes, which limit their applicability. In this study, we present an RO3306 block-and-release strategy to selectively enrich cell populations at defined mitotic stages without compromising cell viability or disrupting their progression to mitotic exit. This approach provides a reliable method for studying mitotic events with high temporal resolution. Furthermore, by preserving mitotic integrity, it offers a valuable framework for investigating the molecular mechanisms of cell division and the processes driving genomic instability in human cells.

## 1. Introduction

The cell cycle is a tightly regulated sequence of events that ensures the formation of two genetically identical daughter cells. In eukaryotic cells, DNA replication occurs during the S phase, while chromosome segregation (mitosis) and cytoplasmatic division (cytokinesis) occur later in the M phase. These two major phases are separated by two gap phases: G1, during which the cell grows and prepares for DNA synthesis, and G2, where it prepares for mitosis [1].

Progression through each phase of the cell cycle depends on the proper completion of the previous one. Checkpoints regulate this unidirectional process by functioning as DNA surveillance mechanisms, ensuring accuracy, and preventing the accumulation of genetic errors during cell division [2]. Eukaryotic cells have three major checkpoints in their cell cycle: the G1/S checkpoint (restriction point), the G2/M DNA damage checkpoint, and the spindle assembly checkpoint (SAC), which occurs during mitosis. These control mechanisms are mediated by cyclin-dependent kinases (CDKs) and their regulatory cyclin subunits, along with other regulatory proteins. During cell cycle progression, CDKs are activated upon binding to their specific cyclin partner, whose expression levels fluctuate throughout the cell cycle, ensuring the precise temporal activation of each CDK [3,4]. In mammalian cells, different CDK–cyclin complexes regulate distinct stages of the cycle: CDK4/CDK6–Cyclin D complex activation drives the progression through G1, whereas the transition from G1 to S is controlled by the CDK2–Cyclin E complex. The progression through S and G2 is regulated by the CDK2–Cyclin A complex and, finally, the entry into M phase is modulated by the CDK1–Cyclin A/B complexes [5].

The study of the cell cycle typically relies on experimental cell models. However, the asynchronous nature of cultured cells limits the analysis of specific stages. Since each cell progresses independently, the population consists of cells at different phases at a given time, limiting the representation of each stage and making it challenging to obtain sufficient samples and conclusive results for specific mechanisms or events. Cell cycle synchronization is an effective strategy to address this limitation. This approach aims, with varying degrees of success, to generate a homogeneous population of cells that progress through the cycle in a coordinated manner, enriching the population at the targeted phase. Cell synchronization methods fall into two main categories: non-chemical and chemical approaches. Non-chemical synchronization methods include physical fractionation, cellular environment modifications (such as serum starvation), and genetic manipulation [6]. While these approaches have fewer side effects, they are less effective and less phase-specific. In contrast, chemical synchronization relies on inhibitors that block key proteins involved in cell cycle progression. This approach is more efficient and precise, making it widely used in cell cycle research [7]. However, to ensure robust synchronization and minimize side effects, several critical factors, including inhibitor selection, dosage, and exposure time, must be carefully controlled.

Over the years, a wide variety of synchronization protocols using chemical inhibitors have been developed. To arrest cells in G1, Palbociclib, a CDK4 and CDK6 inhibitor, and thymidine, which inhibits DNA synthesis, are widely used [8]. For mitotic synchronization, inhibitors such as Colcemid, Nocodazole, or S-Trityl-L-cysteine (STLC) are frequently employed, as they disrupt microtubule dynamics and prevent cells from overcoming the SAC [9,10]. Although these inhibitors are reversible, they present notable drawbacks. For instance, following Colcemid release, only a small proportion of cells successfully proceed through mitosis, and they do so with significantly slower kinetics [10]. On the other hand, Nocodazole treatment results in better cell recovery but significantly increases mitotic defects, including a higher incidence of lagging chromosomes in anaphase and increased frequencies of disorganized and multipolar mitotic spindles [11,12]. Other mitotic inhibitors include MG132, a proteasome inhibitor that arrests cells in metaphase [13,14], and Blebbistatin, which delays anaphase and telophase by inhibiting cleavage furrow ingression [15]. However, the main limitations of using these inhibitors are their insufficient phase-specificity and their mechanism of inducing mitotic arrest by altering the mitotic process itself. To improve phase-specificity, combinatorial approaches involving multiple inhibitors are frequently employed [16]. However, these methods are more time-consuming and introduce further alterations to cell cycle and mitotic progression, representing a major limitation, particularly for genome instability research.

In this study, we present a protocol to synchronize Retinal Pigment Epithelial 1 (RPE-1) cells at distinct mitotic stages using a single treatment with RO3306, a CDK-1 inhibitor [17]. A thorough washout procedure ensures complete drug removal and enables precise control over post-release timing. This approach allows for the synchronization of cells at precise mitotic sub-stages while maintaining cell viability and minimizing time consumption. Additionally, we demonstrate that this method preserves mitotic integrity, as cells arrested at the G2/M boundary successfully progress through mitosis within the expected timeframe after RO3306 release, without affecting mitotic exit.

Building on these findings, our synchronization method effectively enhances the proportion of cells in specific mitotic phases, which are otherwise scarce in asynchronous cultures. This enrichment enables a more detailed analysis of rare events, such as chromosome segregation defects, mitotic spindle abnormalities, and cytokinesis errors, offering a powerful approach for studying the mechanisms driving genomic instability and cancer.

## 2. Results

### 2.1. Enrichment of Mitotic Cells Using an RO3306 Block-And-Release Method

**Figure 1:**
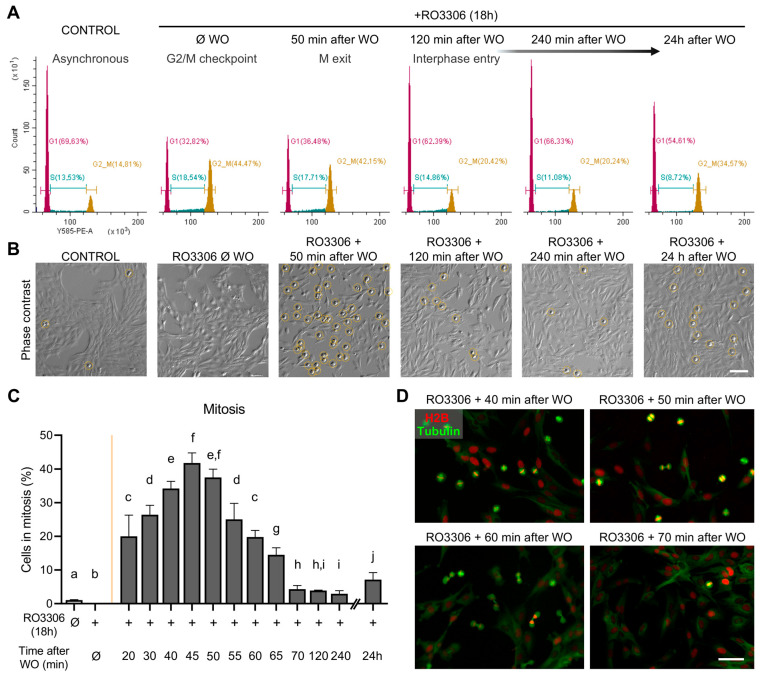
Cell cycle progression following RO3306 block-and-release approach. (**A**) Representative histograms of cell cycle analysis for untreated asynchronous RPE-1 cells (CONTROL) and synchronized samples at different time points after RO3306 washout (WO). Cell cycle distribution was obtained by flow cytometry based on DNA content (propidium iodide staining). (**B**) Representative images of untreated asynchronous RPE-1 cells (CONTROL) and synchronized cells at different time points after RO3306 washout (WO). Yellow circles indicate mitotic cells. Scale bar = 100 µm. (**C**) Frequency of mitotic cells in asynchronous samples of RPE-1 cells (CONTROL) and in synchronized samples at different time points after RO3306 washout (WO). The yellow line indicates the washout time point. *N* = 1000 cells from three independent replicates per condition. Mean and SD are shown (Fisher’s test; different letters denote statistical differences between conditions, *p* < 0.05). (**D**) Representative images of synchronized cells transduced with tubulin-GFP (green) and H2B-RFP (red) at different time points after RO3306 washout (WO). Scale bar = 50 µm.

To assess the utility of an RO3306 block-and-release approach for synchronizing cells in mitosis, we treated RPE-1 cells with 9 µM RO3306 for 18 h and analyzed cell cycle progression following RO3306 release. Flow cytometry-based analysis of DNA content demonstrated that, in an asynchronous population (untreated control), approximately 14% of cells were in the G2/M stage (Figure 1A, Figure S1). In contrast, after 18 h of RO3306 treatment, this percentage significantly increased to 44%, indicating that a substantial fraction of cells was arrested at the G2/M checkpoint. Concurrently, the fraction of cells in G1 decreased substantially, from 70% to 33%. Complementary morphological analysis using phase-contrast microscopy revealed that cells treated with RO3306 for 18 h (RO3306 Ø WO in Figure 1B) underwent noticeable changes in morphology. These cells displayed a more compact and rounded morphology compared to the cells of the untreated sample (CONTROL in Figure 1B), which consisted primarily of elongated, spread-out interphase cells characteristic of an asynchronous culture. This morphological change reflects the G2/M arrest induced by RO3306. In the third image of Figure 1B (50 min after WO), a significant proportion of cells had entered mitosis, as indicated by their more rounded morphology, typical of cells undergoing mitotic entry. Together, these results confirm that the RO3306 treatment effectively arrests approximately half of the cell population at the G2/M checkpoint.

At 2 h post-release, flow cytometry analysis showed that the majority of cells had re-entered the G1 phase (62%; Figure 1A), while only 20% remained in G2/M phases. At this time point, differences compared to the asynchronous population were no longer significant (Figure S1). This trend persisted at 4 h post-release, indicating that the majority of synchronized cells had completed mitosis and continued cycling. By this time, the cell cycle profile closely resembled that of the asynchronous population, further supporting the successful release of the RO3306 blockage and the restoration of normal cell cycle dynamics. At 24 h post-release, flow cytometry analysis confirmed that the cell cycle had been fully re-established (Figure 1A). Interestingly, a significant increase in the proportion of cells in the G2/M phase was observed compared to the control population (Figure S1), suggesting that the cells had completed one full cycle and maintained some degree of synchronization for 24 h.

To obtain insights into mitotic progression upon RO3306 washout, a detailed analysis of the first two hours after release was performed. As illustrated in Figure 1C, the frequency of mitotic cells steadily increased over time after RO3306 release, peaking around 40–45 min post-washout, when most cells are in metaphase (Figure 1D, panel 40 min washout). The real-time monitoring of histone H2B and tubulin revealed that, from this point onward, cells initiate the mitotic exit, progressing through metaphase, anaphase, and telophase, ultimately giving rise to two daughter cells (Video S1). Consequently, the proportion of mitotic cells in culture gradually declined, reaching minimal levels by 70 min post-release, when only a small subset of cells remained in mitosis (mean percentage of mitotic cells at 70 min post-washout = 4.32%; Figure 1C). These findings indicate that most synchronized cells successfully enter and complete mitosis within approximately one hour after release from the G2/M checkpoint.

Morphological analysis and viability assessment at 7 and 24 h after RO3306 release further confirmed that 99.5% of cells remained viable following the applied treatment (Figure S2; Chi-square test, *p* > 0.05). Therefore, the RO3306 block-and-release synchronization method effectively synchronizes cells in mitosis without disrupting the progression of this particularly sensitive cell cycle stage or compromising long-term cell viability.

### 2.2. Controlled Enrichment of Mitotic Populations

Synchronization with RO3306 enables the enrichment of mitotic cell populations within a precisely defined time window. Upon release, RO3306-synchronized RPE-1 cells transition from G2/M arrest and complete mitosis within minutes. Given the inherently brief nature of mitosis, this synchronization method ensures that a high proportion of cells are in mitosis simultaneously. However, due to the complexity of mitotic progression, dissecting the underlying mechanisms often requires isolating specific sub-stages. Therefore, we next sought to determine whether the RO3306 block-and-release approach could selectively enrich cell populations at distinct mitotic sub-stages, thereby facilitating a more precise analysis of mitotic events.

#### 2.2.1. Morphological Classification of Mitotic Cells

**Figure 2:**
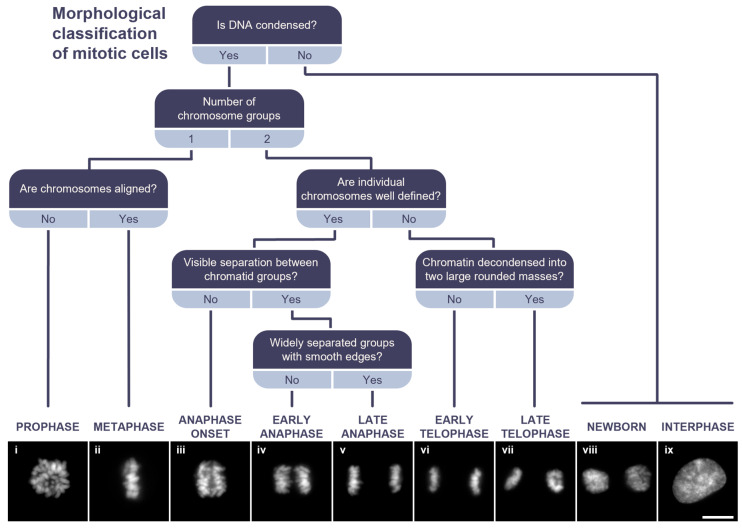
Morphological classification of mitotic cells. Decision tree for classifying mitotic sub-stages based on DAPI staining. Panels (i–ix) show representative images of cells in mitosis and interphase. Scale bar = 10 µm.

Chromosome dynamics during mitosis involve morphological changes that are observable under a microscope. Pioneering work by Fleming in the late 1800s [18], who first illustrated chromosome structure, laid the foundation for the morphological classification of mitotic stages—an approach that has since evolved and remains fundamental in the study of cell division. Thus, using an asynchronous sample as a reference, we constructed a decision tree based on the key morphological features of each mitotic sub-stage (Figure 2). The initial step in identifying mitotic cells involves recognizing those with a higher degree of chromatin condensation compared to interphase cells. Then, the first decision point is determining the number of chromosome groups present. If a single condensed chromosome mass is observed, the cell is classified as being in an early mitotic stage, either prophase (exhibiting varying degrees of chromatin condensation; Figure 2, panel i) or metaphase (Figure 2, panel ii), where chromosomes are aligned along the equatorial plate. Conversely, the presence of two distinct groups of chromatids indicates later mitotic stages, as sister chromatids are moved apart to opposite cell poles, and the cell is preparing for cell division. To further refine classification, the degree of chromatin condensation in cells with two chromatid groups is assessed. If individual chromosomes can still be distinguished, meaning that their contours remain visible, the cell is classified as being in anaphase. In contrast, cells with decondensed or less well-defined chromatin are identified as being in telophase.

For a more precise classification of anaphase cells, the spatial separation between chromatid groups is evaluated. If two chromatid groups are present but no visible separation is observed, the cell is classified as being at the onset of anaphase (Figure 2, panel iii). If a clear separation is visible but the chromatid groups remain closely spaced with irregularly contoured edges, the cell is categorized as early-anaphase (EA; Figure 2, panel iv). In contrast, late-anaphase (LA) cells exhibit widely separated chromatid groups with smooth edges (Figure 2, panel v).

Finally, telophase classification is based on the degree of chromatin decondensation and the spatial distribution of chromatid groups. Early-telophase (ET) cells display moderately decondensed chromatin with distinguishable chromosome boundaries and chromatid groups positioned further apart in two stretched regions (Figure 2, panel vi). In late telophase (LT), chromatin decondensation becomes more pronounced, and chromosome contours lose definition due to the reformation of the nuclear envelope around each chromatin mass, resulting in two rounded structures (Figure 2, panel vii). Thus, based on morphological criteria, mitotic cells can be classified into seven well-defined categories.

#### 2.2.2. Mitosis on Demand: Precise Timing for the Enrichment of Specific Mitotic Sub-Populations

Once the mitotic classification framework was established, we next analyzed RPE-1 cells synchronized using the RO3306 block-and-release method. Following RO3306 washout, cells were fixed at 5- to 10 min intervals, and classified according to the proposed decision tree.

**Figure 3:**
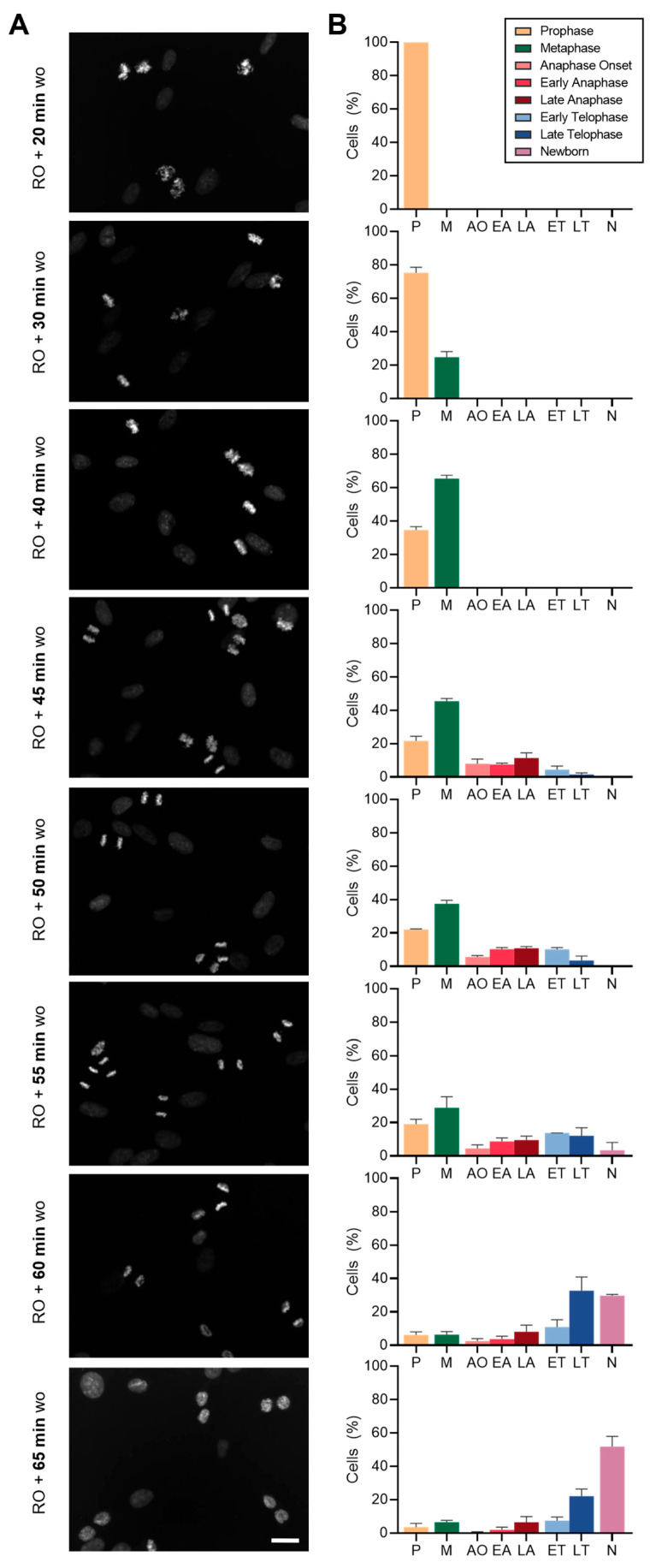
Evaluation of precise timing to enrich specific mitotic sub-populations upon RO3306 release. (**A**) Representative images of DAPI staining at precise time points after RO3306 washout (*WO*). Scale bar = 20 µm. (**B**) Quantification of the percentage of cells at each mitotic sub-stage (*p* = prophase; M = metaphase; AO = anaphase onset; EA = early anaphase; LA = late anaphase; ET = early telophase; LT = late telophase; N = newborn cells). *N* = 250 cells from three independent replicates per condition. Mean and SD are shown.

As shown in Figure 3, at 20 min post-washout, all mitotic cells (100%) were in prophase, indicating a synchronized mitotic entry. By 30 min post-washout, 24.7% of mitotic cells had progressed to metaphase, with this fraction increasing steadily to 65.4% by 40 min. A significant transition occurred at 45 min post-washout, as one-third of mitotic cells (32.9%) had initiated mitotic exit. This transition occurred rapidly between 45 and 55 min post-washout, with a continuous increase in the proportion of cells advancing from early anaphase onset to late telophase. Specifically, at 45 min, 27.0% of mitotic cells were in anaphase, while 6.0% had reached telophase. By 55 min post-washout, the percentage of cells in telophase increased significantly to 25.8%, highlighting the rapid nature of the mitotic exit.

At approximately 60 min post-washout, the first newborn cells—defined as two masses of decondensed chromatin with visible nucleoli but lacking the typical morphology and size of an interphase cell (Figure 2, panel viii versus panel ix)—began to emerge. By 65 min post-washout, newborn cells accounted for 51.7% of the mitotic population, while only 10.0% of the dividing cells remained in the earlier stages of prophase and metaphase. These results suggest that mitotic progression following RO3306 release follows a clear and orderly sequence, with a swift transition from anaphase to telophase and the appearance of newborn cells approximately 60 min after release.

To obtain enriched fractions of cells at specific mitotic sub-stages, it is essential to select the optimal time point when the highest proportion of cells is in the desired stage. While these time points should be precisely determined for each cell type, general guidelines can be established for RPE-1 cells. For prophase-enriched populations, samples should be collected between 20 and 30 min post-RO3306 release. To obtain a higher fraction of metaphase cells, the optimal time point is around 40 min post-release. At later time points, cells begin exiting mitosis, making 50 min post-release ideal for studying late mitotic events such as chromosome segregation. For investigations focused on telophase, sample collection should be extended to approximately 60 min post-release. Finally, to examine the fate of daughter cells following mitotic division, cells should be tracked from 60 min onward.

While this synchronization method is already widely used in many laboratories for culturing synchronized cells, our study offers a more refined protocol, providing detailed guidance on the critical steps to achieve optimal results. In addition, in the subsequent subsection, we will highlight the potential applications of this approach in the study of genomic instability, demonstrating its suitability for investigating the molecular mechanisms behind this phenomenon. By offering both a precise protocol and practical examples of its use, our study contributes valuable insights to the ongoing research in this field.

### 2.3. Applications of RO3306 Synchronization and Precise Washout Protocol

Given the usefulness of the RO3306 block-and-release procedure in enriching cell fractions at specific mitotic sub-stages, we highlight several research applications. Mitotic events play a critical role in determining the fate of daughter cells, making this method invaluable for studying cell division and the mechanisms behind genomic instability. By precisely enriching cells at distinct mitotic phases, our approach enables a focused analysis of chromosome segregation dynamics.

#### 2.3.1. Enrichment of Metaphase Cells for Preparing Chromosome Spreads

Chromosome instability (CIN) is a major form of genomic instability in human cancers [19] and is observed in over 90% of solid tumors [20]. It is primarily associated with defects in chromosome segregation during cell division, leading to numerical chromosomal imbalances (aneuploidy) and, in some cases, unstable structural chromosome abnormalities. These abnormalities can be detected using FISH or immunofluorescence, among other techniques. Moreover, CIN levels are often assessed in metaphase spreads, where chromosomes reach their highest degree of condensation, allowing for more accurate and reliable analysis.

To enrich the proportion of cells in metaphase for chromosome spread preparation, we applied a double synchronization approach. First, RPE-1 cells were synchronized at the G2/M boundary using RO3306. Upon release, microtubule polymerization was inhibited with Colcemid. Since previous data indicate that most RO3306-synchronized cells complete mitosis within an hour, applying Colcemid at this point ensures that the majority of cells are arrested in metaphase. Afterward, we proceeded with cell fixation to obtain metaphase spreads for cytogenetic analysis.

**Figure 4:**
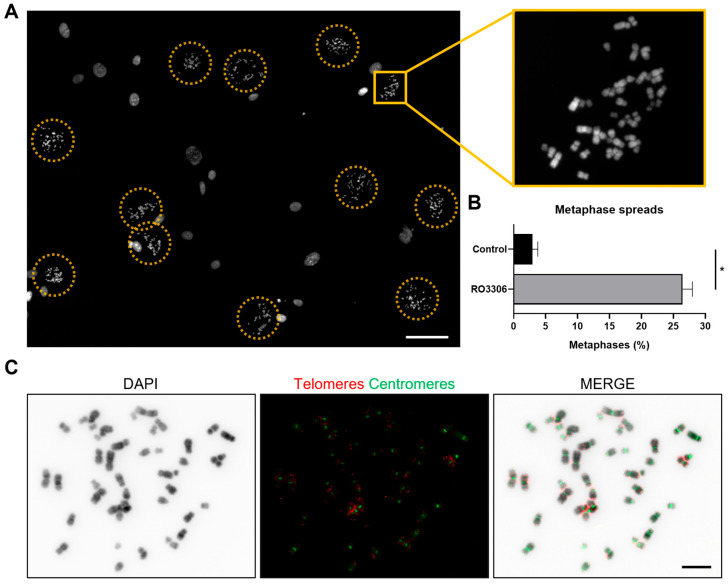
Metaphase spreads with enriched fractions of cells in metaphase. (**A**) Overview of metaphase spreads from RPE-1 cell line after the RO3306 block-and-release approach, followed by Colcemid treatment. Cells were counterstained with DAPI. Yellow circles indicate metaphases. The inset shows a higher magnification of a metaphase. Scale bar = 50 µm. (**B**) Frequency of cells in metaphase in RO3306 untreated RPE-1 cells (*Colcemid*) and in cells double-synchronized with RO3306 and Colcemid. *N* = 1000 cells from three independent replicates per condition. Data are presented as mean and SD (Fisher’s test, * *p* < 0.0001). (**C**) Representative images of telomeres (red) and centromeres (green) in a metaphase spread of synchronized RPE-1 cells, combined with DAPI reverse banding pattern. Scale bar = 10 µm.

This approach significantly increased the proportion of metaphase cells relative to the total cell population, from 2.9% in asynchronous samples to 27.0% in RO3306-synchronized samples (Figure 4A,B; Fisher’s test, *p* < 0.0001). Moreover, the quality of the obtained metaphase spreads was optimal, and the samples were successfully used for FISH techniques, as shown in Figure 4C, where telomere and centromere FISH probes were applied. Therefore, by combining the DAPI reverse banding pattern with the position of the centromere and telomeres of each chromosome, a precise cytogenetic analysis can be conducted.

#### 2.3.2. Enrichment of Cells at Mitotic Exit for Chromosomal Segregation Studies

Chromosome segregation errors during mitosis can arise from multiple defects, including aberrant mitotic spindles, improper microtubule–kinetochore attachments, and irregular cytokinesis [21]. These defects contribute significantly to numerical chromosomal instability, further exacerbating genome instability and cellular dysfunction. Synchronizing cells at specific mitotic sub-phases facilitates a more detailed examination of these mechanisms.

**Figure 5:**
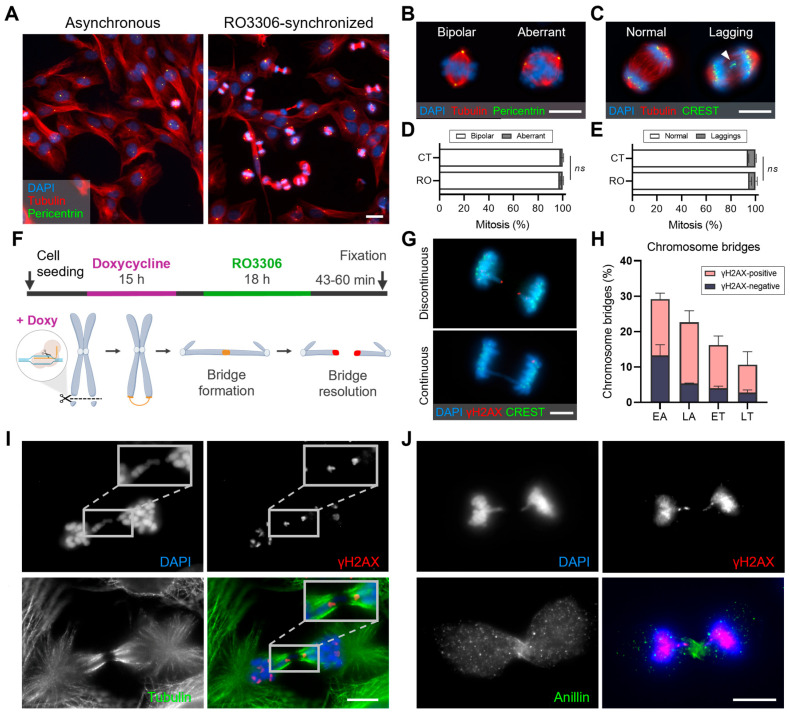
Enrichment of cells at the mitotic exit for the study of chromosome segregation. (**A**) Overview of the immunodetection of α-tubulin (red) and pericentrin (green) in asynchronous and RO3306-synchronized RPE-1 cells. DNA is counterstained with DAPI (blue). Scale bar = 20 µm. (**B**) Representative images of bipolar and aberrant mitotic spindles. Scale bar = 10 µm. (**C**) Representative images of α-tubulin (red) and CREST (green) immunodetection combined with DAPI staining (blue). The arrowhead indicates a lagging chromosome. Scale bar = 10 µm. (**D**) Frequency of bipolar and aberrant (non-bipolar) mitotic spindles relative to the total number of mitotic cells in asynchronous RPE-1 cells (CT) and 50 min after RO3306 release (RO). *N* = 400 mitotic cells from two independent asynchronous samples and 1500 mitotic cells from three independent synchronized samples. Mean and SD are shown (Fisher’s test; *ns*, *p* > 0.05) (**E**) Frequency of lagging chromosomes relative to the total number of cells at the mitotic exit from asynchronous RPE-1 samples (CT) and 50 min after RO3306 release (RO). *N* = 100 cells at the mitotic exit from two independent asynchronous samples and 200 cells at the mitotic exit from three independent synchronized samples. Mean and SD are indicated (Fisher’s test; *ns*, *p* > 0.05. (**F**) Timeline scheme illustrating the experimental design for inducing chromosome bridges in RPE-1 CRISPR/Cas9 sgRNA cell lines. Cells were treated with doxycycline for 15 h (purple line), followed by the RO3306 block-and-release procedure (green line) to enrich the frequency of cells at the mitotic exit. Below the timeline is a schematic representation of the CRISPR/Cas9 approach used to generate chromosome bridges. Doxycycline-induced Cas9 generates breaks at sgRNA-targeted sites in the subtelomeric regions of specific chromosomes (sgRNA for chromosomes 1, 2, or 3). The resulting DNA ends (highlighted in yellow) can re-ligate with their sister chromatid, forming a dicentric chromosome. Upon chromosome bridge resolution, DNA double-strand breaks (γH2AX-positive, red) are generated. (**G**) Representative images of continuous and discontinuous chromosome bridges (DAPI, blue) exhibiting γH2AX (red) and CREST (green) signals. Scale bar = 5 µm. (**H**) Frequency of continuous (γH2AX-negative) and discontinuous (γH2AX-positive) chromosome bridges in cells during early anaphase (EA), late anaphase (LA), early telophase (ET), and late telophase (LT). *N* = 150 cells for each mitotic phase from three independent sgRNA cell lines. Mean and SD are shown. (**I**,**J**) Representative images of chromosome bridges in irradiated MCF10A cells synchronized using the RO3306 approach. Chromosome bridges are identified with DAPI (blue), and DNA double-strand breaks are immunodetected with γH2AX (red). The contractile ring is visualized using (**I**) α-tubulin (green) or (**J**) anillin (green), a key protein involved in cytokinesis that facilitates the correct position of the contractile machinery. Scale bar = 10 µm.

The immunodetection of specific proteins in samples enriched for cells at mitotic exit (50–55 min after RO3306 washout; Figure 5A) enables the detailed analysis of CIN-related structures. For example, the loss of mitotic spindle bipolarity can be assessed through the specific labeling of tubulin and pericentrin (Figure 5B). Additionally, by applying double staining with CREST (to detect kinetochores) and tubulin, the presence of lagging chromosomes can be evaluated (Figure 5C). Lagging chromosomes are a well-known consequence of improper kinetochore–microtubule attachment, leading to chromosomes that fail to migrate correctly during anaphase and remain between the two segregating chromosome masses, ultimately resulting in erroneous chromosome segregation. Importantly, the synchronization methodology does not inherently induce these structures, as confirmed by comparative analysis with asynchronous samples (Figure 5D,E). Therefore, this approach provides a reliable and controlled system to investigate CIN-associated mitotic defects, enabling the precise characterization of chromosome mis-segregation events under different experimental conditions.

The RO3306 block-and-release approach is also particularly useful for studying the resolution of chromosomal bridges during mitosis. Chromosome bridges result from mechanisms contributing to CIN, such as defective DNA damage response, telomere dysfunction, replicative stress, and errors in cell division. Moreover, the resolution of chromosome bridges determines the extent of acquired genomic instability, ultimately affecting cell viability and tumorigenic potential.

To investigate chromosome bridge resolution during mitosis, a doxycycline-inducible CRISPR/Cas9 system was employed to generate bridges with defined characteristics under precise temporal control. In this approach, single-guide RNAs (sgRNAs) target the subtelomeric region of a specific chromosome, where the Cas9 enzyme induces a double-strand break (Figure 5F). Cells were fixed at different time points after RO3306 release to enrich populations at distinct mitotic stages, and dicentric chromosomes resulting from end-to-end fusions were analyzed. Our findings demonstrate that CRISPR/Cas9-generated chromosome bridges primarily resolve during early mitotic exit. Nearly 30% of RPE-1 cells in early anaphase display a bridge of the targeted chromosome. Notably, more than half of these bridges are discontinuous, with γH2AX foci flanking the break site, indicating early-anaphase bridge breakage (Figure 5G,H). The remaining intact bridges resolve in late anaphase, and by telophase, continuous chromosome bridges are observed in fewer than 5% of cells. These bridges may persist into the next interphase [22,23] or resolve during cytokinesis (Figure 5I,J). The synchronization of cells at 60 min post-release has been particularly informative for observing chromosome bridge resolution in the context of cytokinesis. The presence of γH2AX foci on bridges at this stage supports the hypothesis that resolution may also occur during this stage. These observations highlight the utility of mitotic synchronization strategies for dissecting the molecular mechanisms that lead to chromosome instability.

Overall, our study emphasizes the importance of precise temporal control in investigating mitotic defects and CIN-associated phenotypes. Enriching for mitotic sub-phases facilitates the study of the molecular mechanisms underlying both normal and aberrant chromosome segregation, thereby advancing the understanding of genome stability maintenance in human cells.

## 3. Discussion

Our results show that the RO3306 block-and-release synchronization method effectively arrests cells at the G2/M boundary and enables their synchronized progression through mitosis upon drug release. In recent years, other protocols using chemical inhibitors have been developed to study mitotic progression by enriching for the mitotic cell fraction. For example, treatments with nocodazole or STLC (S-Trityl-L-cysteine) induce prometaphase arrest, and their reversibility allows cells to proceed through mitosis following drug release [24]. However, these inhibitors disrupt proper bipolar mitotic spindle formation [14,22], interfering with the earliest stages of mitosis. As a result, the entire mitotic process may be compromised, limiting the study of the mechanisms underlying cell division [11,25]. In contrast, RO3306 treatment arrests cells before mitotic entry, allowing them to progress through mitosis once released. This approach, therefore, facilitates the monitoring and analysis of the dynamics and key events that take place during mitosis.

In addition to enriching the overall mitotic population, the RO3306 block-and-release method offers a more precise approach by selectively enriching cell fractions at specific mitotic sub-phases. While several procedures have been developed for this purpose, most existing methods rely on combining multiple inhibitors [15,26,27], which require time-consuming procedures, often exceeding 24 h. Furthermore, these approaches induce mitotic errors, including chromosome segregation defects and alterations in mitotic dynamics. In contrast, our results show that RO3306 synchronization allows for precise temporal control, enabling the enrichment of cell fractions at specific mitotic sub-phases with just a single inhibitor, by simply adjusting the post-release timing. The main advantage of this method is its ability to synchronize cells in less than 24 h while preserving mitotic integrity.

Contrary to studies suggesting that RO3306 may induce cell death, chromosome segregation defects, and changes in the duration of mitosis [27,28,29], our findings show that under our specific RO3306 treatment conditions and followed by a thorough washout procedure, neither cell viability nor mitotic progression was compromised. We observed no significant increase in mitotic abnormalities, such as lagging and bridging chromosomes, or aberrant mitotic spindles, indicating that the procedure itself does not introduce mitotic defects. Synchronized cells successfully completed mitosis and transitioned into the subsequent interphase of the cell cycle. Notably, we observed a fraction of cells that progressed synchronously to the next mitosis, demonstrating the ability of this approach to maintain long-term synchronization. Furthermore, the timing of mitotic phases and the overall duration of mitosis were consistent with results from other studies and with the normal mitotic timing observed in unsynchronized RPE-1 cells [30]. These findings confirm that our synchronization procedure does not interfere with mitotic dynamics.

In conclusion, by precisely enriching cell populations at specific mitotic stages without compromising cell viability or cell cycle progression, this synchronization approach facilitates the investigation of the mechanisms underlying each specific phase of mitosis. This strategy offers a powerful framework for in-depth studies of cell cycle progression, mitotic dynamics, and genomic instability, paving the way for further research into a wide range of cellular processes essential for both fundamental and applied biomedical research. While our study focuses on RPE-1 cells, we recognize that synchronization efficiency may vary across different cell types. Further validation in additional cell lines will be necessary to strengthen the proof of principle and assess the broader applicability of this approach in diverse cellular contexts.

## 4. Materials and Methods

### 4.1. Cell Culture

Telomerase-immortalized RPE-1 cells expressing Cas9 under a doxycycline-inducible promoter (a gift from Iain Cheesman’s Laboratory) were cultured in DMEM:F12 (Biowest, Riverside, UK) supplemented with 10% tetracycline-free fetal bovine serum (FBS) (FBS-TET-12A, Labclinics, Barcelona, Spain). When indicated, RPE-1 cells were genetically modified using lentiviral particles. MCF10A mammary epithelial cells were cultured in DMEM:F12 supplemented with 5% horse serum, 20 ng/mL epidermal growth factor (EGF), 500 ng/mL hydrocortisone, 100 ng/mL cholera toxin, and 0.01 mg/mL insulin. For lentiviral production, HEK 239T cells were cultured in minimal essential medium (Biowest, Riverside, UK) supplemented with 10% FBS (S181B-500, Labclinics, Barcelona, Spain). All media was supplemented with 1% Penicillin/Streptomycin (100 U/mL, Biowest, Riverside, UK) and 1% GlutaMAX (Thermo Fisher Scientific, Waltham, MA, USA), and cells were maintained in a humidified incubator at 37 °C and 5% CO_2_.

### 4.2. Lentiviral Particle Production and Transduction

Lentiviral particles were produced using HEK 293T cells at approximately 70% confluency. Transfections were performed with 1 µg/mL of total DNA plasmid (Table 1) following the procedure available on Trono Laboratory’s website. The DNA concentration was adjusted to achieve a ratio of 4:3:1 (transfer plasmid/psPAX2/pMD2). Following 48 h, the supernatant was collected, concentrated using centrifugal filters (UFC910008, Merck, Kenilworth, NJ, USA), and stored at −80 °C. Cells were transduced overnight with a medium containing 4 µg/mL polybrene and the lentiviral particles. To enrich the population of transduced fluorescent cells, fluorescence-activated cell sorting was performed using a BD FACSDiscover™ S8 Cell Sorter (BD Biosciences, Franklin Lakes, NJ, USA). To select cells with the shp21/Rb or with sgRNAs, antibiotic selection with 2.5 µg/mL blasticidin (15205, Merck, Kenilworth, NJ, USA) for two weeks was conducted.

### 4.3. Cell Cycle Synchronization and Cell Treatments

Cells were synchronized at G2/M boundary by inhibiting CDK-1 with 9 µM RO3306 (SML0569, Sigma-Aldrich, St. Louis, MO, USA) for 18 h. Drug removal was performed by washing the cells seven times with PBS and then the cells were immediately incubated at 37 °C and 5% CO_2_. For live-cell experiments, the cells were monitored over time. Fixed samples were obtained after fixation with 4% paraformaldehyde at precise time points after RO3306 washout.

To obtain enriched populations of cells in metaphase for chromosome spread preparation, the cells were treated immediately after RO3306 release with 0.05 µg/mL Colcemid for 1 h. Subsequently, a hypotonic shock was performed using 0.075 M KCl for 13 min at 37 °C, followed by fixation in methanol/acetic acid (3:1). Cell suspensions were dropped onto slides to obtain metaphase spreads. 

To generate chromosome bridges under controlled conditions, Cas9 expression was induced with 1 µg/mL doxycycline (631311, Clonetech, Mountain View, CA, USA) for 15 h [23]. Then, the cells were rinsed seven times with PBS and returned to the incubator for 5 h before RO3306 block-and-release treatment.

When indicated, exponentially growing MCF10A were exposed to 2.5 Gy of γ-rays using an IBL-437C R-137 Cs irradiator at a 5.10 Gy/min dose rate. Following irradiation, the cells were returned to the incubator for 24 h and then synchronized using the RO3306 block-and-release approach.

### 4.4. Cell Cycle

The analysis of DNA content with flow cytometry was performed as described by Darzynkiewicz and collaborators (2001) [32]. Cells were harvested by trypsinization, fixed in 70% ethanol, and stored at −20 °C for at least 2 h. Afterward, the cells were stained with propidium iodide (PI) staining solution (0.1% Triton-X100 in PBS, 0.2 mg/mL DNase-free RNase A, and 0.02 mg/mL of PI) and incubated at room temperature for 30 min. Fluorescence intensity was measured using a CytoflexLX (Beckman Coulter, Brea, CA, USA) equipped with a 561 nm laser and a 585/42 BP filter. Data were analyzed using CytExpert software v2.4 (Beckman Coulter, Franklin Lakes, NJ, USA).

### 4.5. Live/Dead Assay

LIVE/DEAD assay (Invitrogen, Waltham, MA, USA) was performed following the manufacturer’s instructions. Cells were incubated with the staining solution (0.5 µg/mL calcein and 3 µM ethidium homodimer-1 (EthD-1) in medium) for 30 min at 37 °C. Images of the stained samples were acquired using an inverted microscope Olympus IX71 (Olympus, Tokyo, Japan). Live cells, stained in green (calcein-positive) and dead cells, stained in red (EthD-1-positive), were quantified using the Cell Counter plugin in ImageJ software v1.54k [33].

### 4.6. Immunofluorescence

After fixation, cells were permeabilized for 15 min with 1× PBS/0.5% Triton-X-100 solution, blocked for 15 min with 1× PBS/0.5% BSA/0.15% glycine, and subsequently incubated overnight at 4 °C with primary antibodies (listed in Table 2). After incubation, the cells were rinsed three times with 1× PBS/0.1% Tween 20, followed by 1 h incubation at room temperature with secondary antibodies (listed in Table 2). Afterward, the cells were rinsed three times with 1× PBS/0.1% Tween 20, briefly rinsed with distilled water, and progressively dehydrated in ethanol. Finally, samples were mounted on glass microscope slides with Vectashield mounting medium (Vector Laboratories, Inc., Burlingame, CA, USA) containing 0.25 µg/mL of 4′,6-diamidino-2-phenylindole (DAPI). Visualization and image acquisition were performed on an Olympus BX61 epifluorescence microscope (Olympus Tokyo, Japan) equipped with a CV-M4+CL camera (JAI, København, Denmark) and CytoVision software v3.6 (Applied Imaging, San Jose, CA, USA). 

### 4.7. Fluorescence In Situ Hybridization

Slides with metaphase spreads were treated with pepsin/HCl solution at 37 °C for 90 s, post-fixed with formaldehyde-MgCl2 for 10 min, and dehydrated in ethanol. The hybridization mix was prepared containing a pancentromeric PNA probe conjugated with FITC (AAACACTCTTTTTGTAGA) (Panagene, Daejeon, Republic of Korea) and a pantelomeric PNA probe conjugated with Cy3 (CCCTAA) (PE Byosystems, Foster City, CA, USA). DNA on the slides was denatured at 80 °C for 90 s and subsequently hybridized with the hybridization mix at room temperature for 2 h. After hybridization, the slides were washed using 70% formamide and Tris/NaCl/Tween 20 buffer, dehydrated, and counterstained with Vectashield mounting medium containing 0.25 μg/mL DAPI. Sample visualization and imaging were conducted using an Olympus BX61 microscope (Olympus, Tokyo, Japan) and CytoVision software v3.6 (Applied Imaging, San Jose, CA, USA). Image analysis was performed using ImageJ.

### 4.8. Live-Cell Imaging

For time-lapse experiments, RPE-1 cells transduced with tubulin-GFP and H2B-RFP were seeded onto glass-bottom culture dishes (µ-Dish 35 mm, high Glass Bottom, ibidi GmgH, Gräfelfing, Germany). The day after seeding, the cells were synchronized with RO3306, and image acquisition was initiated at 45 min after RO3306 washout. Confocal images were obtained using a Zeiss LSM980 laser scanning confocal microscope (Zeiss, Oberkochen, Germany) equipped with Diode 488 nm and DPSS 561 nm lasers. Using Airyscan mode and a 25× objective (25×/0.8 LD LCI Plan-Apochromat Imm Korr DIC M27 Oil), a total of 17 z-stacks at 1.2 µm intervals were acquired every 4 min for 2 h. During image acquisition, cells were maintained under controlled conditions (37 °C and 5% CO_2_), and the definite focus approach was used to prevent focus drift. Images were processed with ZEN Blue software v2.3 (Zeiss, Oberkochen, Germany).

### 4.9. Statistics

Statistical analyses and graph plotting were performed using GraphPad Prism 8 (GraphPad Software version 8.0.1, San Diego, CA, USA). Data are presented as mean ± SD. Details of the statistical test applied are provided in the figure legends. *p*-values < 0.05 were considered statistically significant.

## Figures and Tables

**Table 1:** List of plasmids.

Plasmid Name	Targeting Sequence + PAM	Antibiotic Resistance	Vector Type	Reference
shp21/Rb	-	Blasticidin	Lentiviral	Pellman’s Laboratory
H2B-RFP	-	-	Lentiviral	Addgene #26001
Tubulin-GFP	-	-	Lentiviral	Addgene #64060
sgChr1	TGACGTTGGACAGCCGCTGGAGG	Blasticidin	Lentiviral	Anglada et al., [31]
sgChr2	ATATTAAGGGCTCCCCGTCGGGG	Blasticidin	Lentiviral	
sgChr3	AGGGCTGGTCACCATTCAAGAGG	Blasticidin	Lentiviral	
psPAX2	-	-	Packaging	Addgene #12260
pMD2.G	-	-	Envelope	Addgene #12259

**Table 2:** List of antibodies.

Antibody	Host	Reference	Working Dilution
PRIMARY ANTIBODIES			
Anti- α-Tubulin	Mouse	Sigma-Aldrich (St. Louis, MO, USA), T5168	1:1000
Anti-Pericentrin	Rabbit	Abcam (Cambridge, UK), ab4448	1:1500
Anti-CREST	Human	Antibodies Incorporated (Davis, CA, USA),15-234	1:50
Anti- γH2AX (Ser139)	Mouse	Millipore (Burlington, MA, USA), 05-636, clone JBW301	1:1000
Anti- γH2AX (Ser139)	Rabbit	Abcam, ab81299, clone EP854(2)Y	1:500
Anti-Anillin	Rabbit	Abcam, ab99352	1:1000
SECONDARY ANTIBODIES			
Anti-Mouse Cyanine Cy™3	Goat	Jackson ImmunoResearch Inc. (West Grove, PA, USA), 115-165-146	1:500
Anti-Mouse Alexa Fluor^®^488	Goat	Jackson ImmunoResearch Inc., 115-545-205	1:800
Anti-Rabbit Alexa Fluor^®^488	Goat	Thermo Fisher Scientific (Waltham, MA, USA), A-11034	1:500
Anti-Rabbit Alexa Fluor^®^594	Goat	Thermo Fisher Scientific A-11037	1:500
Anti-Human FITC	Goat	Antibodies Incorporated, 52-241-0100	1:100

## Data Availability

The original contributions presented in this study are included in the article/Supplementary Materials. Further inquiries can be directed to the corresponding author.

## Supplementary Materials

The following supporting information can be downloaded at: https://www.mdpi.com/article/10.3390/ijms26104951/s1.
