# Supplementary material for: Controlled Exit from the G2/M Checkpoint in RPE-1 Cells Using RO3306: Enrichment of Phase-Specific Cell Populations for In-Depth Analyses of Mitotic Events
Source: Int J Mol Sci. 2025 May 21;26(10):4951. doi: 10.3390/ijms26104951 (PMC12112338; doi:10.3390/ijms26104951)
Supplement: Supplementary file 1 [file ijms-26-04951-s001.zip › ijms-3508947-figures.docx]

**Controlled Exit** from G2/M Checkpoint: Enrichment **of Phase-Specific Cell Populations for In-Depth analyses of Mitotic Events**

**Teresa Anglada*, Núria Pulido-Artola, Marina Rodriguez-Muñoz and Anna Genesca***


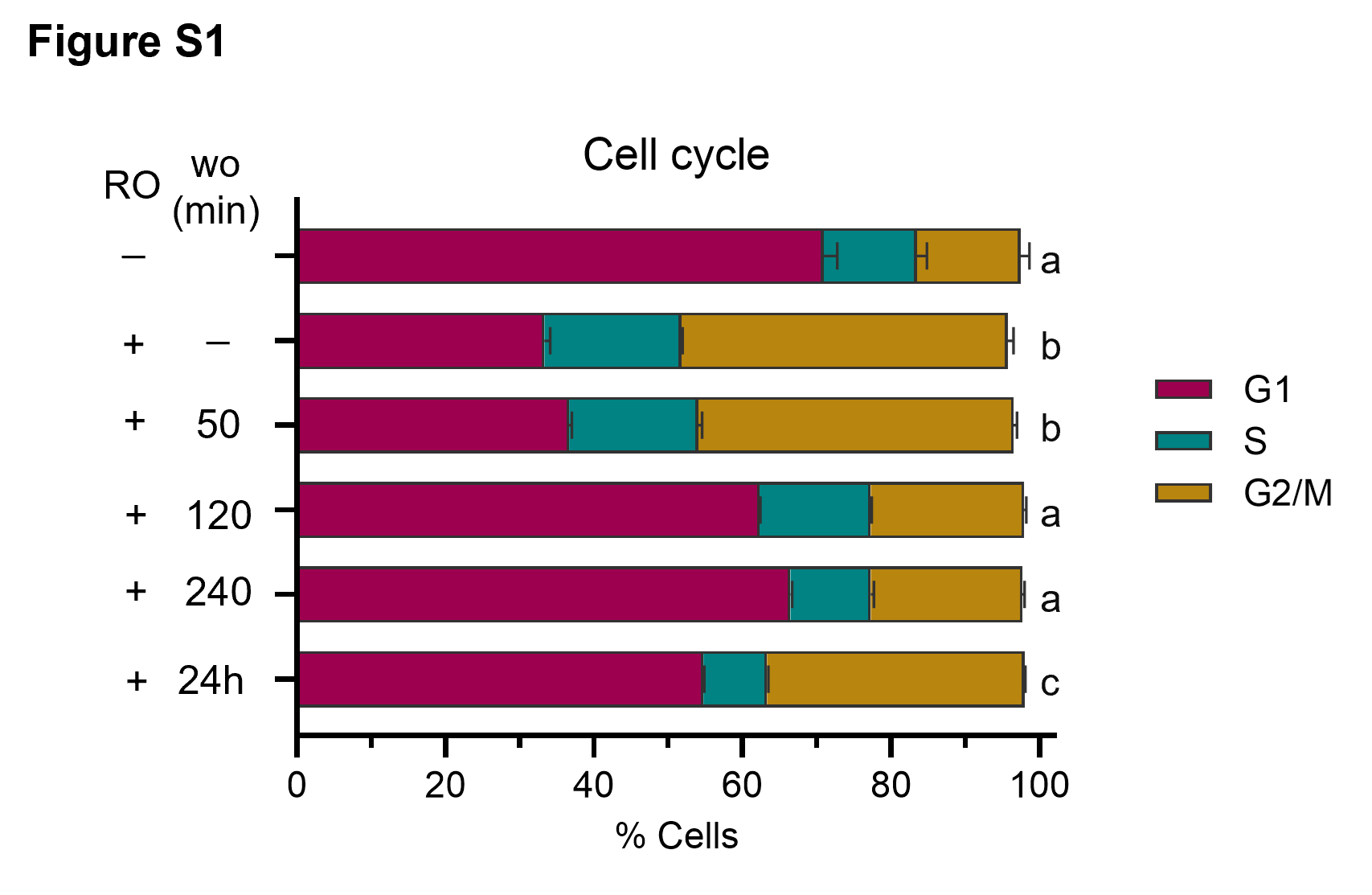


Figure S1: Cell cycle analysis obtained by PI staining. Quantification of cells in G1, S and G2/M phases in untreated samples and at different time points after RO3306 washout (wo). *N* = 20000 cells from two independent replicates per condition. Statistical differences in the G2 population are indicated (multiple t-test with False Discovery Rate approach; different letters indicate statistically significant differences between conditions, *p* < 005).


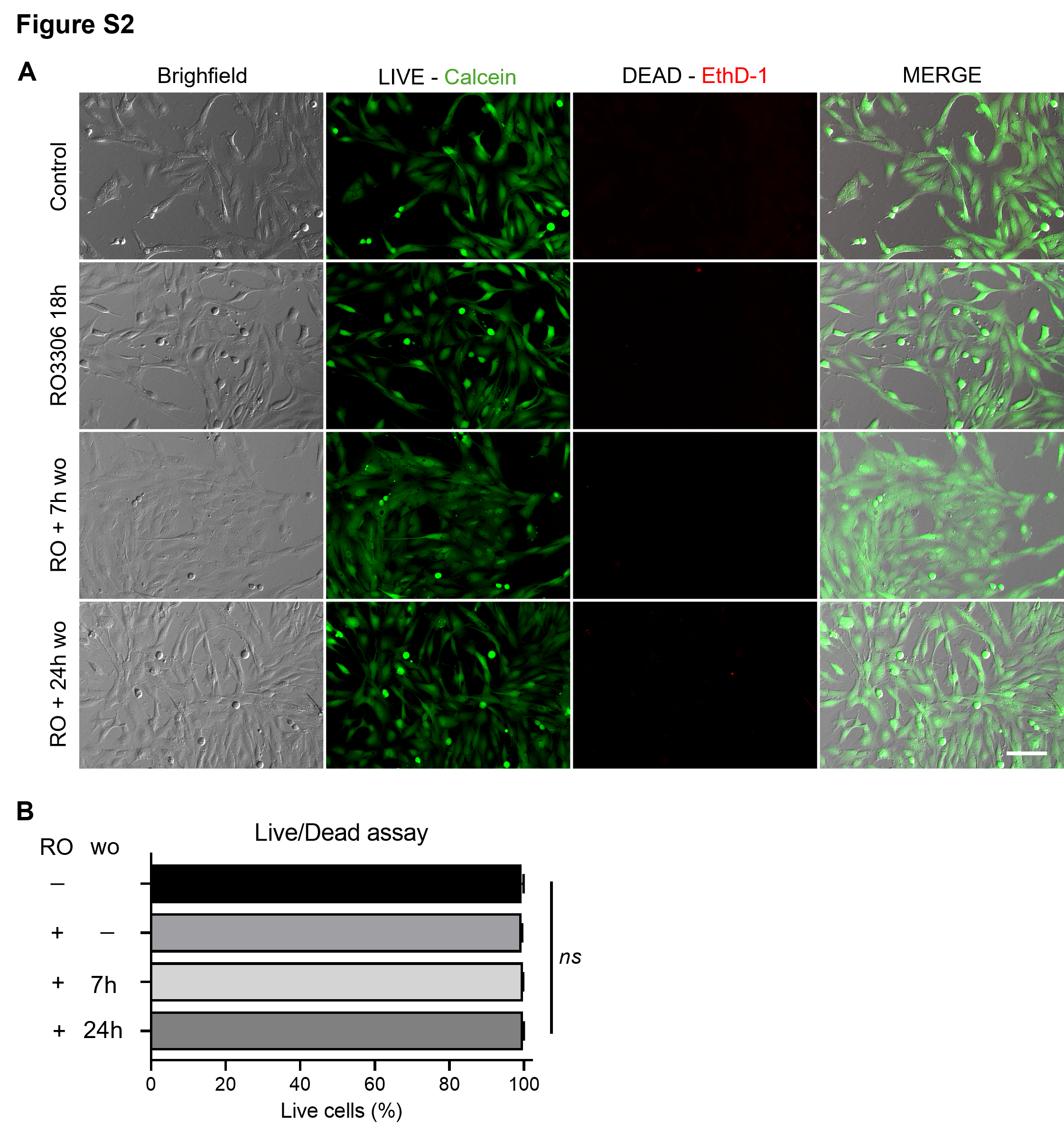


Figure S2: Live/Dead assay to assess cell viability following RO3306 treatment. (A) Representative images of calcein and ethidium homodimer-1 (EthD-1) staining in an asynchronous sample, after an 18-hour RO3306 treatment, and at 7 or 24 hours post-RO3306 washout (wo). Scale bar = 100 µm. (B) Quantification of calcein-positive cells over total cell population. A minimum of 1000 cells from three independent replicates were analyzed. Mean and SD are indicated (Chi-square test; *ns,* *p* > 0.05).
